# Exposure to violence and socioeconomic deprivation in susceptibility to nitrogen dioxide on term infant birthweight in New York City

**DOI:** 10.1186/s12940-025-01189-0

**Published:** 2025-05-31

**Authors:** Richard V. Remigio, Heather H. Burris, Jane E. Clougherty

**Affiliations:** 1 Department of Global, Occupational, and Environmental Health, School of Public Health, University of Maryland, College Park, College Park, MD USA; 2 Urban Health Collaborative, Dornsife School of Public Health, Drexel University, Philadelphia, PA USA; 3 Division of Neonatology, Department of Pediatrics, Children’s Hospital of Philadelphia, University of Pennsylvania Perelman School of Medicine, Philadelphia, PA USA; 4 Department of Environmental and Occupational Health, Dornsife School of Public Health, Drexel University, Philadelphia, PA USA

## Abstract

**Introduction:**

Air pollution has been associated with adverse birth outcomes, with variation by socioeconomic position (SEP). However, it remains unknown which aspects of lower SEP – comprised of myriad physical and psychosocial stressors – may best explain observed pollution susceptibilities. Building upon previous studies that estimated joint associations of air pollution and socioeconomic deprivation on term birth weight in New York City (NYC), this study seeks to identify specific social stressors underlying that relationship.

**Methods:**

We examined records for 243,853 term births in NYC from 2007–2010. Residence-specific pregnancy-average NO_2_ was estimated using NYC Community Air Survey (NYCCAS) and EPA regulatory data. Twenty-six community social stressor indicators were tested as modifiers of NO_2_-birthweight associations in linear mixed-effects models, adjusting for particulate matter (PM_2.5_), individual-level maternal characteristics, and other covariates. In sensitivity analyses, we also examined non-linear interactions between continuous NO_2_ and census-tract level violence and deprivation terms.

**Results:**

Consistent with previous work, a 1-IQR (6.2 ppb) increase in average prenatal NO_2_ exposure was associated with a 12.6 (SE = 2.7)-gram decrease in term birthweight.We observed similar values in independent models for most stressors related to violent crime or SEP and significantly lower birthweights with higher stressor exposures.

In models of effect modification, however, we found that – despite lower average birthweights in high-stressor communities – NO_2_-birthweight associations were *weaker* in higher-stressor communities, particularly for violence-related stressors. For example, in the highest-quartile communities for assault, a 1-IQR increase in NO_2_ exhibited a decrement of only 7.3 g, on average, compared to 16.9 g in the lowest-assault quartile (*p* = .01 trend across quartiles). Exposures to non-violent stressors were not significantly associated with lower birthweights, nor modified observed NO_2_-birthweight associations.

**Conclusions:**

We found significantly lower term-infant birthweights with higher NO_2_ or community stressors. Counter to hypotheses, however, in communities with very high stressor exposures (esp. violent crimes), despite lower overall birthweights, associations for NO_2_ were *weaker* than in low-stressor communities.

Our results suggest a possible saturation effect in stress-pollution interactions, wherein very high stressor exposures appear to overwhelm any effects of pollution. In addition, we observed stronger effects for violent crimes, in relation to other social stressors.

**Supplementary Information:**

The online version contains supplementary material available at 10.1186/s12940-025-01189-0.

## Introduction

Urban air pollution has been associated with negative birth outcomes including lower birthweights [1, 2], higher rates of preterm birth [3], neurocognitive impairments [4], and infant mortality [5]. Lower socioeconomic position (SEP) has also been associated with both adverse birth outcomes [6] and greater susceptibility to pollution [7]. It remains unknown, however, which aspects of lower SEP – a construct that includes a host of physical and psychosocial stressors (e.g., housing quality, crime, food insecurity, etc.) – may best explain observed variation in pollution susceptibility by SEP.

Associations between urban air pollution and birth outcomes may be mediated, in part, via systemic oxidative stress [8, 9], or inflammation [10], leading to placental insufficiency. There has been substantial variation in observed relationships between air pollution and fetal growth, however, potentially attributable to differences in exposure measurement or misclassification [11], co-pollutant adjustments [12], incomplete adjustments for confounding, or differential exposure–response relationships across populations. Appropriately accounting for SEP is a particular challenge, as SEP is often spatially correlated with air pollution [13, 14], and thus may confound measures of association, while common biologic pathways related to inflammation, immune, and neuroendocrine dysregulation may facilitate synergistic effects, and variation in true effects of pollution across SEP subgroups [15, 16].

Low area-level SEP has been associated with adverse pregnancy outcomes after accounting for individual-level SEP. Separately, studies have reported greater susceptibility to air pollution in communities of lower SEP, with effects on respiratory disease and asthma [17, 18], cardiovascular disease [19, 20], birthweight [21, 22], and neurocognitive development [23, 24]. Notably, the key “causal constituents” underlying SEP-related susceptibility remain undefined [25], as SEP includes a host of chemical and non-chemical exposures accumulating and interacting over the life course. Growing evidence suggests that chronic stressors, which may explain a substantial portion of this SEP-related susceptibility [26], given evidence of immune, endocrine, and metabolic dysregulation associated with chronic psychosocial stress (i.e., allostatic load) [27].

Racial disparities in birth outcomes including preterm birth and low birthweight are well-documented, and may be attributable to substantial differences in social and environmental exposures between non-Hispanic Black and non-Hispanic White individuals [28]. In a commentary discussing the future of perinatal epidemiology and pre-term birth cohorts [29], Ness and colleagues described the need to disentangle complex interactions among social and physical exposures—which requires large and diverse samples, detailed information on environmental exposures and community stressors, and innovative analytic strategies to address persistent spatial confounding among correlated exposures [30].

In this study, we explored a more comprehensive set of community stressors associated with lower SEP, to consider their independent and synergistic associations, with full-gestation nitrogen dioxide (NO_2_) exposures, on term birthweight, in an urban setting. We focused on NO_2_ as a marker of traffic-related air pollution, with substantial spatial variation within New York City (NYC) [29]. As our health outcome, we focused on birthweight among term births, as an indicator of fetal growth with important life-course and population health implications [31]. Here, we build on a prior study of fine-scale spatio-temporal air pollution exposures and birth outcomes in NYC [32, 33], which reported significant associations between fine-scale NO_2_ with term birthweight [32], and stronger NO_2_-birthweight associations in census tracts in the highest and lowest quartile of a socioeconomic deprivation index (SDI) [7].This work incorporated previously-generated data on area-level air quality [32] and disaggregated community stressors that were used to develop composite social indicators [7] to measure their association with birth weight in NYC. We focused on analyzing the main effect and modifying role of individual community stressors (*n* = 26) in the association between NO_2_ and birthweight.

## Methods

### Study population

We examined records for approximately 380,000 live births among residents of the five boroughs of NYC in 2008–2010, merged with patient-level data from the New York State Department of Health Statewide Planning and Research Cooperative System (SPARCS), which includes all licensed healthcare facilities. Data were restricted to full-term (37—42 weeks gestation) singleton births with no congenital anomalies, no self-reported maternal smoking during pregnancy, and with complete residential address and covariate data. Exclusion criteria for implausible clinical values and fixed cohort bias [34] in this population are previously detailed [32, 35].

### Air pollution exposure assessment

Residence-specific nitrogen dioxide (NO_2_) and fine particulate matter (PM_2.5_) exposure estimates were created using 100-m resolution spatial surfaces from the NYC Community Air Survey (NYCCAS) and daily EPA regulatory data and averaged each live birth based on the gestation period. We estimated near-residence (300-m) air pollution exposures between December 2008 and December 2010. NYCCAS monitoring and modeling methods are detailed elsewhere [29, 36]. Briefly, NYCCAS used spatial saturation monitoring to estimate multiple combustion-related pollutants across 150 locations in four seasons over two years. Also, two-week integrated samples were collected at street-level (10–12 feet) each season.

Exposure estimates were temporally adjusted using regulatory monitoring data to match individual-level gestation periods. Births were geocoded to the mother’s residential address at delivery. NYCCAS pollution concentration surfaces were used to estimate near-residence exposure as the mean concentration within a 300 m radial buffer [33]. In keeping with previous analyses, we examined full-gestation average NO_2_, as modified by social stressors, and adjusted for spatio-temporal PM_2.5_ as a co-pollutant [7].

### Term birthweight outcome and covariates

Birth records were linked through NYC’s Bureau of Vital Records to NYC residents. After applying the exclusion criteria, we restricted our sample population to 243,345 births. Term birthweight as an outcome was examined as a continuous variable. We adjusted for individual covariates associated with fetal growth, including averaged prenatal PM_2.5_ exposure, maternal age, race/ethnicity [White, Black, Hispanic, or Asian, cross-classified by United States (US)- vs. foreign-born status], maternal age, education (< 9, 9–11, 12, 13–15, 16, or > 16 years), Medicaid status (yes/no), pre-pregnancy body mass index (BMI), receipt of prenatal care (yes/no), year of conception, parity (number of prior live births), infant sex, and gestational age (in weeks). To adjust for temporal trends in pollution, we also adjusted for conception year and season, as previous [32].

### Community social stressor indicators

We linked individual maternal residences to administrative data representing 23 individual community social stressors, derived from federal, state, and city data sources, and broadly representing stressors in six domains [i.e., crime and violence; physical disorder, noise disruption, SEP, and access to health and mental health care]. We then re-aggregated indicators from multiple geographies (e.g., police precinct, census tract) to the United Hospital Fund (UHF) area (*n* = 34), as in Shmool et al. [37]. Three additional indicators are composite variables from a factor analysis of the 23 stressors, which revealed highly correlated factors characterized by: violence and physical disorder (Factor 1), crowding and poor resource access (Factor 2), and complaints related to air pollution or noise (Factor 3). Each indicator is shown in Table 1.

**Table 1:** Community stressor indicators at UHF-level, adapted from [38]. *SR* denotes self-reported variables

Stressor domain	Administrative indicator	Variable name	Data Source
Crime & Violence	Felony larceny crimes	Larceny	New York Police Department (NYPD)
	Felony murder and non-negligent manslaughter	Murder	NYPD
	Felonious assault	Assault	NYPD
	Felony burglary	Burglary	NYPD
	Felony robbery	Robbery	NYPD
	Perceived lack of neighborhood safety (self-report) (SR)	Safety	DOHMH Community Health Survey (CHS)
	Substantiated cases of Child Abuse/Neglect	Abuse	NYC Administration of Child Services
Physical Disorder	Air quality complaints	Air_Compln	NY State Department of Environmental Protection
	Small parks not acceptably clean	Unclean_Parks	Parks Department
	Sidewalks not acceptably clean	Sidewalks	Mayor’s Office of Operations (MOoO)
	Crowding (> 1 occupant/room)	Crowding	US Census American Community Survey (ACS)
	Serious housing violations	Housing_Violation	Dept. of Housing Preservation and Development
Noise disruption	Frequent noise disruption (3 + times/wk) (SR)	Noise_Freq	CHS
	Noise disruption by neighbors (SR)	Noise_Neighb	CHS
	Noise disruption by traffic (SR)	Traffic_Noise	CHS
Income/SEP	Percent < 200% federal poverty line	Poverty	ACS
	Delayed rent or mortgage payment in the past year (SR)	Delay_rent	CHS
	Food stamp program enrollment	Food_Stamps	MOoO
	Unemployed for less than 1 year	Unemployment	ACS
Access to Healthcare	No Insurance coverage (SR)	No_Insurance	CHS
	Went without needed medical care (SR)	No_Med_Care	CHS
	Public health insurance enrollment	Public_Insur	MOoO
	Without personal care provider (SR)	No_Doctor	CHS
Composite Indicators			
Factor 1	Spatial factor characterized by violence and physical disorder	F1	[38]
Factor 2	Spatial factor characterized by crowding and low resource access	F2	[38]
Factor 3	Spatial factor characterized by noise and air pollution complaints	F3	[38]

For comparability, all stressors were coded such that higher values were hypothesized to result in greater risk. We examined variation in birthweight by interquartile range (IQR) of each stressor, testing each as a direct predictor of birthweight (continuous variables). To test as potential modifiers of NO_2_-birthweight associations (interaction analyses), we categorized stressors into quartiles.

### Statistical methods

#### Mixed effects regression

We used a linear mixed-effects approach to estimate the independent and interactive effects for NO_2_ and each social stressor on birthweight. We adjusted for spatio-temporal gestation-averaged PM_2.5_ and covariates, and used a random effect to account for participants within UHFs. The model takes the form shown in Equation 1, where ${Y}_{ij}$ is the birthweight outcome for the *j*th person in the *i*th UHF spatial unit, ${\beta }_{0}$ is the intercept and grand mean, ${b}_{i}$ is a random intercept change for each UHF, gestation-mean NO_2_ concentration is denoted as ${X}_{ij1}$, ${X}_{ij2}$ is an categorical social stressor, ${X}_{ijp}$ include covariates, and ${\varepsilon }_{ij}$ is the error term.

Equation 1:   

$${Y}_{ij}={\beta }_{0}+{b}_{i}+{\beta }_{1}{X}_{ij1}+{\beta }_{2}{X}_{ij2} +{\beta }_{3}{X}_{ij1}{X}_{ij2}+\sum_{p=4}^{14}{\beta }_{p}{X}_{ijp}+{\varepsilon }_{ij}$$

Each social stressor was tested separately as a main effect (continuous variable) adjusted for NO_2_ and covariates, and as a categorical modifier of NO_2_-birthweight associations in interaction models, using the lowest quartile of each stressor as the referent category. We tested for linear trends across quartiles, using two-sided tests and alpha of 0.05.

### Sensitivity Analyses

#### Assumption of linearity, and model specification

 Before finalizing the modeling approach and testing interactions, we tested for non-linearity in the NO_2_-birthweight relationship by fitting an array of models using non-linear formulations of NO_2_ [i.e., natural splines with varying numbers of knot points (1 to 5 knots, and unspecified)], both overall and within quartiles of each social stressor. We also examined non-linear model formulations within varying ranges (subsets) of NO_2_ exposures (i.e., including and excluding outliers, 10—90 th percentile, 25 th—75 th percentile, by stressor quartile) using generalized additive mixed models (GAMM) with a random effect for level of aggregation (UHF or census tract).

We tested the robustness of our results to additional specifications in our modeling methods – including the exclusion of a random effect for UHF in generalized additive models (GAM) models with and without interactions by community social stressors, and various categorizations for stressor modifiers (tertiles, quartiles).

We explored non-linear interactions between continuous NO_2_ and census tract-level indicators of violent crime (assault and total violent crime per 10,000 residential population) and SEP (poverty rate and a socioeconomic deprivation index (SDI)—a composite indicator of tract-level material deprivation [7, 54].

Finally, we examined the robustness of model adjustment by SDI, to account for effects of other co-occurring stressors.

## Results

Among 243,345 live births in NYC, the majority (90.3%) of measured birthweights ranged between 2,500 to 3,999 g. Average prenatal NO_2_ was 26.8 ppb (SD = 5.3 ppb) (Table 2). The majority (64.1%) of births occurred at 39 to 40 weeks gestation to individuals between the ages of 25 and 35 (53%), with normal pre-pregnancy BMI (54.3%). The plurality of individuals reported their race and ethnicity as self-reported foreign-born Hispanic (21.9%) followed by US-born White (19.4%). Summary statistics for social stressor indicators are presented in Supplemental Table 1.

**Table 2:** Study population characteristics, *n* (%) or mean (SD)

		Mean (SD)	*N* (%)
Term birthweight (g)	< 1500	87 (0.04)	
	1,500–2499	6,388 (2.6)	
	2,500–3,999	219,694 (90.3)	
	≥ 4,000	17,176 (7.1)	
Air pollution exposures and social stressor	Prenatal NO_2_ (ppb)	26.8 (5.3)	
	Prenatal PM_2.5_ (µg/m^3^)	11.8 (1.9)	
	Social Deprivation Index (SDI)	−0.10 (0.71)	
Race/Ethnicity	US-born White		47,137 (19.4)
	Foreign-born White		22,890 (9.4)
	US-born Black		29,145 (12.0)
	Foreign-born Black		23,812 (9.8)
	US-born Hispanic		30,270 (12.4)
	Foreign-born Hispanic		53,195 (21.9)
	US-born Asian		2,898 (1.2)
	Foreign-born Asian		33,998 (14.0)
Infant Sex	Male		124,470 (51.1)
	Female		118,875 (48.9)
Maternal age (years)	< 20		16,048 (6.6)
	20- < 25		50,500 (20.8)
	25- < 30		64,690 (26.6)
	30- < 35		64,364 (26.4)
	35- < 40		37,168 (15.3)
	≥ 40		10,575 (4.3)
Maternal education	< 9 yrs		19,711 (8.1)
	9–11 yrs		42,702 (17.5)
	12 yrs (High school)		58,154 (23.9)
	13–15 yrs		53,247 (21.9)
	16 yrs (BA/BS)		39,713 (16.3)
	> 16 yrs		29,818 (12.3)
Medicaid status	No		94,540 (38.9)
	Yes		148,805 (61.1)
Pre-pregnancy BMI	< 18.5 (Underweight)		13,424 (5.5)
	18.5—24.9 (Normal)		132,221 (54.3)
	24.9— 29.9 (Overweight)		57,718 (23.7)
	≥ 30 (Obese)		39,982 (16.4)
Prenatal care received	No		1,271 (0.5)
	Yes		242,074 (99.5)
Parity	0		113,416 (46.6)
	1		71,8383 (29.5)
	2		32,956 (13.5)
	≥ 3		25,135 (10.3)
Gestational age (weeks)	37		19,607 (8.1)
	38		44,901 (18.5)
	39		84,068 (34.5)
	40		72,136 (29.6)
	41		20,956 (8.6)
	42		1,677 (0.7)
Conception year	2007		40,730 (16.7)
	2008		94,038 (38.6)
	2009		90,434 (37.2)
	2010		18,143 (7.5)
Conception season	Dec-Feb		70,089 (28.8)
	Mar-May		49,590 (20.4)
	June-Aug		53,569 (22)
	Sep-Nov		70,097 (28.8)

On average, we observed a 12.9 (SE = 2.7)-gram decrease in newborn birthweight per IQR increase in NO_2_ (IQR = 6.2 ppb) in models adjusting for all covariates except social stressors.

**Fig. 1:**
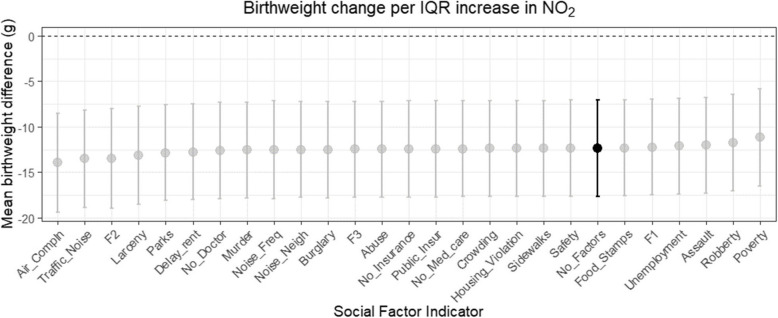
Estimated decrease in term birthweight (g) per IQR NO_2_, after adjusting for covariates and each stressor individually (listed across x-axis). Estimate without any stressor adjustment denoted in black. Models were adjusted for averaged prenatal PM_2.5_ exposure, maternal age, race/ethnicity, maternal age, education, Medicaid status (yes/no), pre-pregnancy body mass index, receipt of prenatal care, year of conception, parity, infant sex, gestational age (in weeks), conception year, and season

In models adjusted for each social stressor (Fig. 1), we observed that NO_2_-birthweight associations were generally robust, though ranged from −14. 7 to −11.4 g per IQR NO_2_, suggesting that NO_2_-birthweight associations were not overly confounded by social stressors. NO_2_-birthweight associations were most attenuated when adjusted for community poverty rates.

**Fig. 2:**
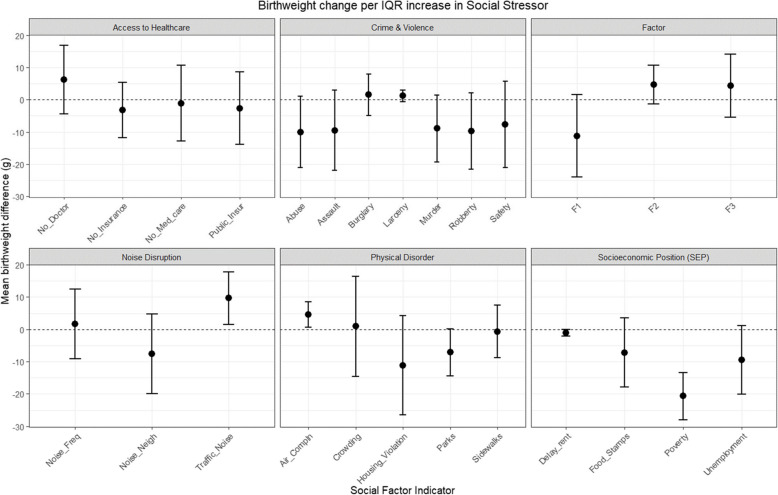
Estimated difference in birthweight per IQR increase in each social stressor, by stressor domain, adjusted for NO_2_, as main effects, and not accounting for interactions. Models were also adjusted for averaged prenatal PM2.5 exposure, maternal age, race/ethnicity, maternal age, education, Medicaid status (yes/no), pre-pregnancy body mass index, receipt of prenatal care, year of conception, parity, infant sex, gestational age (in weeks), conception year, and season

Observing stressor-birthweight associations across the six domains (Fig. 2), we found negative associations with birthweight for most variables related to violent crime (child abuse, assault, murder, robbery, and low perceived safety) or lower SEP (delayed rent, food stamps, poverty, and unemployment), as a main effect. Associations for stressors in all other categories were mixed.

In linear models testing the independent associations between each continuous social stressor and birthweight, adjusted for NO_2_ and covariates, we found a statistically significant decrease in birthweight per IQR increase in UHF-area reported child abuse [β = 12.1 g (95% CI = 1.2–23.0 g)] and the composite Factor 1 (violent crime and physical disorder) [β = 13.3 g (95% CI = 0.7–26.0 g)]. We also observed significantly lower birthweights with poverty [β = 10.8 g (95% CI = 6.8–14.8 g)], unemployment [β = 11.2 g (95% CI = 0.7–21.7 g)], and delayed rent [β = 1.3 g (95% CI = 0.3–2.3 g)]. Finally, we observed significantly lower birthweights with more local unclean parks [β = 7.7 g (95% CI = 0.3–15.1 g)]. When adjusting for NO_2_ and covariates, we observed decreasing birthweights across increasing quartiles of each stressor listed above, and for assault, murder, robbery, housing violations, and reliance on food stamps (*p*-trend < 0.05) (Supplemental Table S3). Residence in the highest quartile for murder, compared to the lowest, adjusting for NO_2_ and covariates, was associated with a decrement of 22.1 g, on average, with consistent decreases in mean birthweight across quartiles (*p*-trend = 0.02) (Supplemental Table S3).

In models examining variation in NO_2_-birthweight associations by each categorical social stressor (interactions), nine of the twenty-six tested stressor indicators (child abuse, assault, food stamps, murder, unclean parks, poverty, robbery, perceived lack of safety, and unemployment) demonstrated potential interactive effects in one more stress or quartiles and five (assault, murder, robbery, perceived lack of safety, unclean parks) displayed consistent NO_2_-birthweight associations, across stressor quartiles (Table 3). In each case, average birthweights were lowest in the highest-stressor quartile (Supplemental Table 2) but observed effects of NO_2_ were weaker with greater stressor intensity.

**Table 3:** Predicted change in birthweight per IQR increase in NO_2_, by stressor quartile, and linear *p*-value for trend across quartiles based on interaction models. Models were also adjusted for averaged prenatal PM_2.5_ exposure, maternal age, race/ethnicity, maternal age, education, Medicaid status (yes/no), pre-pregnancy body mass index, receipt of prenatal care, year of conception, parity, infant sex, gestational age (in weeks), conception year, and season. Significant interactions ( *p* <.05) shown in **bold**

Social Stressor	Administrative indicator	Quartile	Change in birthweight (g) per IQR NO_2_	*p*-trend
**Abuse**	Substantiated cases of Child Abuse/Neglect	Q1	−16.7	0.09
		Q2	−13.3	
		**Q3**	−6.3	
		Q4	−11.0	
Air_Compln	Air quality complaints	Q1	−16.9	0.76
		Q2	−11.9	
		Q3	−19.6	
		Q4	−12.8	
**Assault**	Felonious assault	Q1	−16.9	0.01
		Q2	−18.0	
		**Q3**	−8.5	
		Q4	−7.3	
Burglary	Felony burglary	Q1	−11.9	0.86
		Q2	−10.7	
		Q3	−16.5	
		Q4	−10.9	
Crowding	Crowding (> 1 occupant/room)	Q1	−15.7	0.86
		Q2	−6.8	
		Q3	−12.0	
		Q4	−15.0	
Delay_rent	Delayed rent or mortgage payment in the past year (SR)	Q1	−14.7	0.29
		Q2	−15.0	
		Q3	−11.5	
		Q4	−10.7	
F1	Spatial factor characterized by violence and physical disorder	Q1	−15.4	0.04
		Q2	−15.5	
		Q3	−8.4	
		Q4	−7.3	
F2	Spatial factor characterized by crowding and low resource access	Q1	−15.1	0.26
		Q2	−20.5	
		Q3	−10.1	
		Q4	−13.0	
F3	Spatial factor characterized by noise and air pollution complaints	Q1	−15.4	0.85
		Q2	−9.7	
		Q3	−10.3	
		Q4	−14.3	
**Food_Stamps**	Food stamp program enrollment	Q1	−16.9	0.25
		**Q2**	−8.0	
		Q3	−12.0	
		Q4	−9.9	
Housing_Violations	Serious housing violations	Q1	−15.3	0.03
		Q2	−17.2	
		Q3	−6.6	
		Q4	−8.5	
Larceny	Felony larceny crimes	Q1	−11.5	0.70
		Q2	−15.6	
		Q3	−23.9	
		Q4	−10.6	
**Murder**	Felony murder and non-negligent manslaughter	Q1	−15.1	0.047
		Q2	−11.5	
		Q3	−17.4	
		**Q4**	−3.3	
No_Doctor	Without personal care provider (SR)	Q1	−13.4	0.53
		Q2	−15.4	
		Q3	−11.6	
		Q4	−11.6	
No_Insurance	No Insurance coverage (SR)	Q1	−13.7	0.87
		Q2	−12.9	
		Q3	−9.0	
		Q4	−14.2	
No_Med_Care	Went without needed medical care (SR)	Q1	−13.7	0.12
		Q2	−15.5	
		Q3	−16.9	
		Q4	−5.4	
Noise_Freq	Frequent noise disruption (3 + times/wk) (SR)	Q1	−13.7	0.66
		Q2	−14.9	
		Q3	−13.3	
		Q4	−11.9	
Noise_Neigh	Noise disruption by neighbors (SR)	Q1	−13.6	0.21
		Q2	−19.7	
		Q3	−9.1	
		Q4	−10.9	
**Unclean_Parks**	Small parks not acceptably clean	Q1	−16.9	0.047
		Q2	−13.1	
		**Q3**	−3.2	
		Q4	−9.7	
**Poverty**	Percent < 200% federal poverty line	Q1	−13.7	0.36
		Q2	−8.9	
		**Q3**	−4.1	
		Q4	−11.7	
Public_Insur	Public health insurance enrollment	Q1	−15.0	0.14
		Q2	−12.1	
		Q3	−12.9	
		Q4	−7.2	
**Robbery**	Felony robbery	Q1	−16.9	0.03
		Q2	−14.0	
		Q3	−8.9	
		**Q4**	−6.7	
**Low Perceived Safety**	Perceived lack of neighborhood safety (self-report, SR)	Q1	−14.9	0.02
		Q2	−15.3	
		Q3	−12.3	
		**Q4**	**−4.2**	
Sidewalks	Sidewalks not acceptably clean	Q1	−13.7	0.43
		Q2	−14.0	
		Q3	−11.7	
		Q4	−10.4	
Traffic_Noise	Noise disruption by traffic (SR)	Q1	−15.3	0.21
		Q2	−20.8	
		Q3	−8.9	
		Q4	−12.9	
**Unemployment**	Unemployed for less than 1 year	Q1	−16.6	0.14
		Q2	−9.9	
		Q3	−16.9	
		**Q4**	−6.9	

For example, the average observed birthweight was 35 g lower in communities in the highest quartile for murder rates (mea*n* = 3318 g, SD = 455 g), compared to the lowest quartile (mea*n* = 3353 g, SD = 437 g) (Supplemental Table 2). However, when considering interactions between NO_2_ levels and social factors, in communities with high murder rates, a 1-IQR increase in NO_2_ resulted in only a 3.3-g reduction in birthweight, compared to a 15.1-g reduction in the lowest-murder communities (Table 3). Similarly, while birthweight generally decreased across quartiles of increasing assault (*p*-trend ≤ 0.001; Supplemental Table 2), the observed effect of NO_2_ was dampened from −16.9 g to −7.3 across quartiles.

**Fig. 3:**
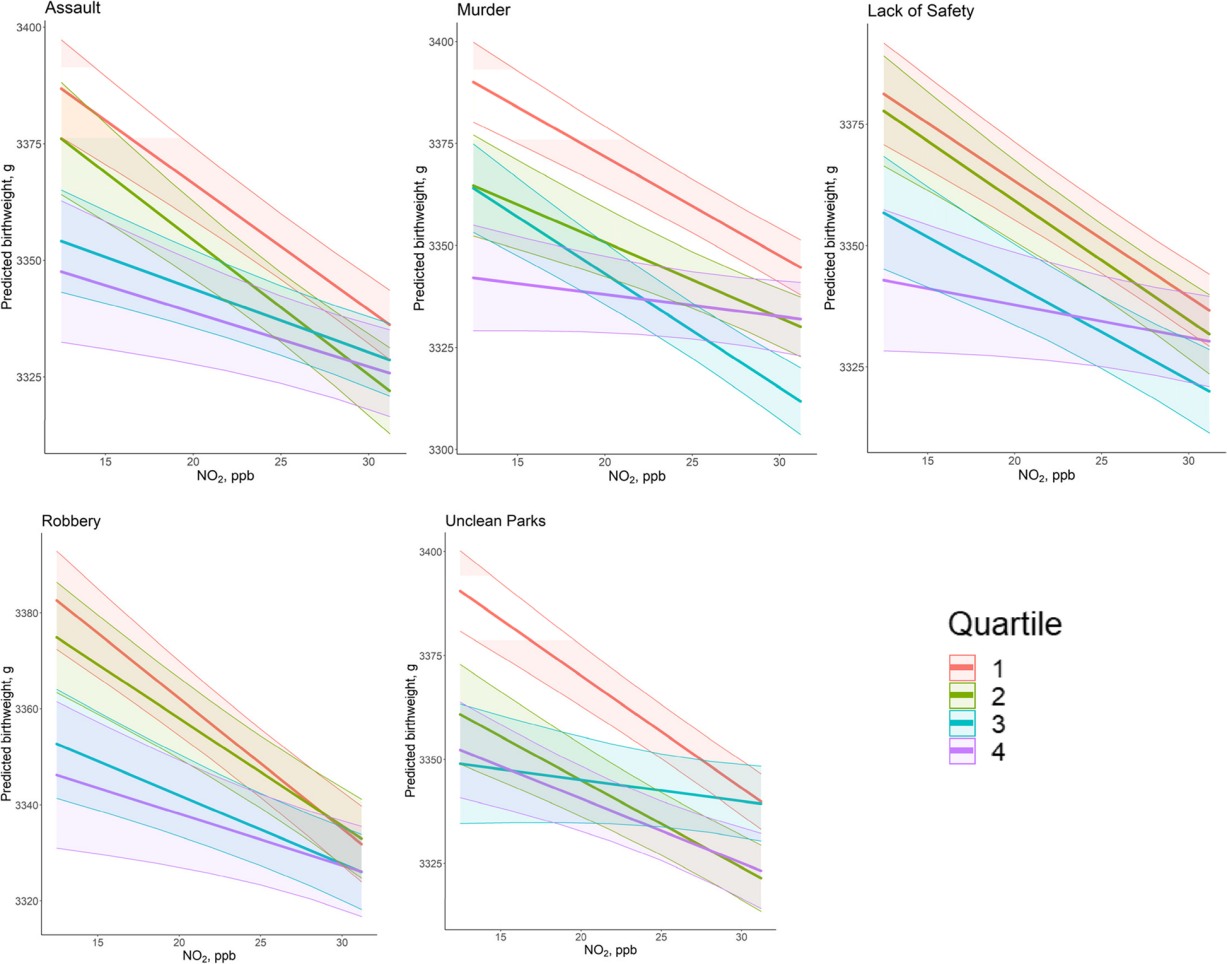
Predicted birthweights and NO_2_-birthweight associations (slopes) across selected community stressor quartiles (1 = lowest stressor exposure; 4 = highest) based on NO_2_ (continuous)-social stressor (categorical) interaction linear mixed effects models. Estimates are shown for the five stressors displaying consistent change in the observed effect of NO_2_ on birthweight, across stressor quartiles, shown for the 10 th to 90 th percentile of NO_2_ exposures

As part of a sensitivity analysis for models that exhibited significant interactions with categorized social stressors as reported in Fig. 3, we mutually adjusted each stressor-NO_2_ interaction model with significant social stressors (child abuse, assault, food stamps, murder, unclean parks, poverty, robbery, perceived lack of safety, and unemployment) as covariates. Overall, we observed point estimate changes across quartiles for each stressor (Supplemental Table S-4). For example, the highest quartile for categorized Murder shifted from 3.3 g reduction to 71.0 g reduction in birth weight per 1-IQR increase in NO_2_.

In all cases, the net effect of pollution and stressors on birthweight was negative. Residents in the highest-stressor quartiles consistently had the smallest infants and the lowest predicted birthweights (Supplemental Table S5. Associations between NO_2_ and birthweight, however, were often dampened in high-stressor quartiles.

We confirmed that NO_2_-birthweight associations were linear overall, and within each quartile of each social stressor, by examining GAMM models stratified by quartile, allowing for varying numbers of knot points. For illustration, Supplemental Fig. 1 depicts the observed linear relationship between NO_2_ and birthweight by quartile of the composite Factor 1 (violence and physical disorder) using natural splines allowing for 5 knots as a sensitivity analysis.

In contrast, excluding UHF as a random effect in GAM models revealed some non-linearity, for a subset of social stressors. For example, in Supplemental Fig. 2, we observe non-linear relationships between NO_2_ and birthweight in the first and second quartile of Factor 1. To account for clustering with UHF areas, we opted to retain the random effect for UHF, supporting a linear analysis.

We found mixed evidence for non-linear modification between continuous NO_2_ and tract-level modifiers. In all cases, however, we did find significant decrements in birthweight with higher violence or SEP.

Adjusting for SDI did not substantially alter coefficient estimates or standard errors for NO_2_ or chronic stressors. We conducted this test to determine if SDI had a more influential role on the modification of the NO_2_-birthweight relationship after adjustments as shown in the Shmool et al. study. The adjustment also did not alter tests for statistical significance in NO_2_–birthweight associations (not shown).

## Discussion

In this work, we conducted a follow-up study that focused on the modifying role of individual social stressors on the association between NO_2_ and birthweight in NYC [7]. There is substantial prior evidence linking air pollution to adverse prenatal and perinatal health [3, 39], and several studies have examined modification by SEP. Though, it remains unknown, however, which specific social stressors explain SEP-related susceptibility. Among singleton births in NYC, we found significantly lower birthweights in higher-traffic pollution, higher-stressor communities. Contrary to hypotheses, however, NO_2_-birthweight associations were weaker in high-stressor communities, particularly for stressors related to violent crime (the composite Factor 1, assault, murder, robbery, and low perceived safety). This result suggests that, although birthweights were lower in high-stressor communities, those low birthweights were not attributable to NO_2_, but were rather more directly related to social conditions themselves. At particularly high levels of some chronic stressors, some ‘saturation’ effect may occur, wherein birthweights are already very low, due to social conditions themselves, and marginal effects of air pollution are no longer observable [15]. In other words, very high stressor exposures may overwhelm any potential effects of pollution, rendering air pollution impacts less evident at very high stressor exposures.

This work provides two key contributions to the literature. First, SEP is a complex construct, including a broad range of social stressors which may act very differently in shaping health outcomes, and disentangling these components is key to identifying effective interventions. Second, interactions are not necessarily linear, nor uniform, as threshold and saturation effects – combined with unique joint distributions between social and environmental exposures in each setting – may lead to very different pollutant-outcome associations along the continuum of any given chronic stressor.

Our results suggest a predominant role for community violence in shaping SEP-related susceptibility, in this urban setting, in keeping with much of our prior work [38, 40–42]. Both qualitative [43, 44] and quantitative [6, 45] work suggest that community violence, and fear thereof, is a critical component of urban SEP-related susceptibility. Prior studies have documented negative impacts of community violence on term birthweight; Masi et al. found up to an average reduction of 43 g in birthweight for a one-unit increase violent crime rate in Chicago, using census tract-level measures of violent crime [6]. Evidence has also linked community violence exposures during pregnancy to greater perceived stress [46], higher preterm birth rates [47], smaller gestational size [6], and pregnancy complications [48].

We also found that one indicator of neighborhood built environment quality (unclean parks) significantly modified NO_2_-birthweight associations. Degraded built environments have been associated with lower birthweights [49] and worse birth outcomes [50], and evidence has shown that, particularly among women, neighborhood physical environments sends important signals regarding safety [51, 52]. As such, this finding may plausibly further support a central role for perceived safety in explaining SEP-related susceptibility among pregnant urban women.

These findings were made possible by revisiting the Shmool et al. study and applying similar methods to characterize the role of individual community stressors as risk factors and effect modifiers. In the Schmool study, a principal component analysis identified seven highly-correlated social stressors: the population percentage of residents with a college degree, unemployment rates, residential crowding, management or professional occupations, households below 200% of the Federal Poverty Level (FPL), households receiving public assistance, and non-White racial composition. These stressors were used to develop an area-level composite index for socio-economic deprivation. In this study, we adopted a more comprehensive and expansive framework to identify individual stressors that significantly influence the relationship between nitrogen dioxide (NO_2_) levels and birthweight. These findings may have implications for developing more targeted and focused interventions that address domains related to socio-economic position, crime and violence, and physical disorder. A major advantage of this work is our access to exhaustive births data throughout NYC, capturing neighborhoods greatly varying in air pollution and social stressor exposures. In addition, we used highly spatially-resolved air pollution exposure estimates, reducing exposure misclassification. Also, multiple spatial predictors for chronic stressors were accessed through publicly available sources specific to a densely populated city. We were able to geocode and link detailed birth data across the study area. Also, we were able to adjust for several traditionally used maternal risk factors related to inter-uterine growth restriction.

There are also some limitations to this work. UHF, as a spatial unit (*n* = 34 across NYC), does not capture very fine-scale neighborhood influences. In tract-level sensitivity analyses, we found significant main effects for violent crime or SDI terms, though mixed evidence for non-linear interactions.

We did not simultaneously adjust for multiple social factors (except for sensitivity-testing using SDI), nor incorporate measures of race-based racial segregation, as doing so could plausibly dampen observed effects of specific social stressors, given the role of historical redlining and segregation in shaping current-day spatial distributions in social stressors. Gestation-averaged NO_2_ concentration was used as an indicator of NO_2_ exposure throughout pregnancy, following prior findings in this data that trimester-specific exposures did not yield meaningfully different results [7]. The analytic dataset is restricted to term infants, as such, associations of exposures with preterm birth could not be assessed. We speculate that restricting term births may bias findings toward the null since severely growth restricted fetuses are often born preterm [53]. Finally, the data is over a decade old, though the observed associations between pollution and birth outcomes, as well as modification by social stressors, remain salient. Future analyses will address several of the limitations above, focusing on that subset of social stressors available at finer geographic scales.

Our results suggest a predominant role for violence-related stressors in explaining SEP-related susceptibility to pollution—in this urban U.S. setting—and point to complex non-linearities in interactions among social stressors and pollutants. Further research in large datasets is needed to elucidate non-linear interactions among social stressors and pollution exposures.

## Supplementary Information

Supplementary Material 1.

## Data Availability

No datasets were generated or analysed during the current study.
