# Supplementary material for: Exposure to violence and socioeconomic deprivation in susceptibility to nitrogen dioxide on term infant birthweight in New York City
Source: Environ Health. 2025 May 31;24:33. doi: 10.1186/s12940-025-01189-0 (PMC12125727; doi:10.1186/s12940-025-01189-0)

**SUPPLEMENTAL MATERIALS**

**Supplemental Table 1.** Summary statistics for social stressors (mean and standard deviation (sd), including quartile divisions (25th percentile (q1), median, and 75^th^ percentile (q3)).

| **Variable Name** | **Variable Description** | **mean** | **sd** | **q1** | **median** | **q3** |
| --- | --- | --- | --- | --- | --- | --- |
| **Child_Abuse** | Substantiated cases of Child Abuse/Neglect | 1.09 | 0.77 | 0.59 | 0.77 | 1.59 |
| **Air_Complnt** | Air quality complaints | 2.2 | 1.77 | 1.23 | 1.47 | 2.23 |
| **Assault** | Felonious assault | 1.26 | 0.64 | 0.75 | 1.15 | 1.75 |
| **Burglary** | Felony burglary | 3.66 | 1.02 | 3.01 | 3.54 | 4.01 |
| **Crowding** | Crowding (>1 occupant/room) | 1.18 | 0.52 | 0.79 | 1.01 | 1.79 |
| **F1** | Spatial factor characterized by violence and physical disorder | 0.05 | 0.64 | -0.57 | -0.04 | 0.43 |
| **F2** | Spatial factor characterized by crowding and low resource access | -0.03 | 1.12 | -0.72 | -0.3 | 0.28 |
| **F3** | Spatial factor characterized by noise and air pollution complaints | 0.2 | 0.83 | -0.43 | 0.14 | 0.57 |
| **Food_Stamps** | Food stamp program enrollment | 1.33 | 0.71 | 0.83 | 1.12 | 1.83 |
| **Hous_Violat** | Serious housing violations | 0.69 | 0.53 | 0.29 | 0.44 | 1.29 |
|  |  |  |  |  |  |  |
| **Larceny** | Felony larceny crimes | 5.94 | 3.69 | 4.52 | 5 | 5.52 |
| **Murder** | Felony murder and non-negligent manslaughter | 1.14 | 0.77 | 0.53 | 0.95 | 1.53 |
| **No_Doctor** | Without personal care provider (SR) | 2.09 | 0.74 | 1.51 | 2.05 | 2.51 |
| **No_Insur** | No Insurance coverage (SR) | 2.54 | 0.96 | 2.03 | 2.45 | 3.03 |
| **No_Med_Care** | Went without needed medical care (SR) | 1.94 | 0.6 | 1.51 | 2.03 | 2.51 |
| **Noise_Freq** | Frequent noise disruption (3+times/wk) (SR) | 2.47 | 0.67 | 1.89 | 2.42 | 2.89 |
| **Noise_Neighb** | Noise disruption by neighbors (SR) | 2.29 | 0.59 | 1.86 | 2.1 | 2.86 |
| **Unclean_Parks** | Small parks not acceptably clean | 2.03 | 0.9 | 1.36 | 1.99 | 2.36 |
| **Public_Insur** | Public health insurance enrollment | 2.03 | 0.72 | 1.62 | 2.08 | 2.62 |
| **Robbery** | Felony robbery | 1.63 | 0.61 | 1.05 | 1.65 | 2.05 |
| **Safety** | Perceived lack of neighborhood safety (self-report) (SR) | 1.08 | 0.6 | 0.58 | 0.99 | 1.58 |
| **Sidewalks** | Sidewalks not acceptably clean | 1.59 | 0.92 | 0.98 | 1.49 | 1.98 |
| **Noise_Traffic** | Noise disruption by traffic (SR) | 3.39 | 0.79 | 2.82 | 3.26 | 3.82 |
| **Unemployment** | Unemployed for less than 1 year | 2.89 | 0.74 | 2.33 | 2.68 | 3.33 |
| **Poverty** | Percent < 200% federal poverty line | 1.32 | 0.63 | 0.81 | 1.33 | 1.81 |

**Supplemental Table S2.** Average observed birthweight stratified by categorical stressors as quartiles.

| **Social Factor Indicator** | **Q1** | **Q2** | **Q3** | **Q4** | *p***-trend** |
| --- | --- | --- | --- | --- | --- |
| **Air_Complnt** | 3319 (453) | 3328 (451) | 3342 (438) | 3358 (442) | < 0.001 |
| **Assault** | 3354 (437) | 3340 (445) | 3327 (451) | 3319 (456) | < 0.001 |
| **Burglary** | 3351 (443) | 3332 (440) | 3328 (452) | 3336 (451) | < 0.001 |
| **Child_Abuse** | 3356 (437) | 3348 (438) | 3317 (454) | 3323 (456) | < 0.001 |
| **Crowding** | 3352 (445) | 3323 (446) | 3324 (454) | 3355 (435) | 0.347 |
| **F1** | 3355 (436) | 3336 (440) | 3333 (453) | 3321 (456) | < 0.001 |
| **F2** | 3332 (445) | 3326 (451) | 3338 (451) | 3352 (438) | < 0.001 |
| **F3** | 3336 (449) | 3330 (446) | 3334 (451) | 3346 (440) | < 0.001 |
| **Food_Stamps** | 3355 (439) | 3329 (441) | 3335 (451) | 3327 (456) | < 0.001 |
| **Hous_Violat** | 3351 (437) | 3343 (448) | 3322 (448) | 3331 (455) | < 0.001 |
| **Larceny** | 3345 (439) | 3323 (452) | 3323 (452) | 3346 (445) | 0.791 |
| **Murder** | 3353 (437) | 3344 (442) | 3329 (453) | 3318 (455) | < 0.001 |
| **No_Doctor** | 3345 (444) | 3339 (448) | 3321 (450) | 3343 (443) | 0.005 |
| **No_Insur** | 3344 (445) | 3334 (448) | 3320 (453) | 3346 (440) | 0.328 |
| **No_Med_Care** | 3346 (438) | 3330 (449) | 3352 (449) | 3315 (450) | < 0.001 |
| **Noise_Freq** | 3336 (449) | 3342 (444) | 3348 (443) | 3321 (450) | < 0.001 |
| **Noise_Neighb** | 3356 (443) | 3335 (447) | 3336 (440) | 3316 (457) | < 0.001 |
| **Noise_Traffic** | 3328 (452) | 3335 (450) | 3345 (441) | 3340 (442) | < 0.001 |
| **Parks** | 3352 (441) | 3328 (452) | 3330 (444) | 3336 (447) | < 0.001 |
| **Poverty** | 3355 (443) | 3334 (447) | 3329 (446) | 3329 (450) | < 0.001 |
| **Public_Insur** | 3353 (445) | 3325 (444) | 3332 (448) | 3337 (450) | < 0.001 |
| **Robbery** | 3355 (440) | 3335 (440) | 3335 (451) | 3319 (456) | < 0.001 |
| **Safety** | 3355 (438) | 3347 (444) | 3318 (448) | 3324 (456) | < 0.001 |
| **Sidewalks** | 3341 (443) | 3333 (446) | 3342 (442) | 3329 (456) | 0.003 |
| **Unemploy** | 3356 (440) | 3348 (441) | 3320 (449) | 3320 (456) | < 0.001 |

**Supplemental Table S3.** Change in average birthweight across quartiles of each social stressor from non-interaction categorical social stressor-birthweight models. P-value is for the significance of the trend in mean birthweight across quartiles. Models were also adjusted for averaged prenatal PM_2.5_ exposure, maternal age, race/ ethnicity, maternal age, education, Medicaid status (yes/no), pre-pregnancy body mass index, receipt of prenatal care, year of conception, parity, infant sex, gestational age (in weeks), conception year, and season. Asterisks denote significance of difference from reference group (Q1). Note: *p<0.05; ** p<0.01; ***p<0.001

| Social Factor Indicator | Q1 | Q2 | Q3 | Q4 | *p*-trend |
| --- | --- | --- | --- | --- | --- |
| Abuse | Ref | -1.9 (-20.9- 17.1) | -24.3 (-43.0- -5.6) * | -17.5 (-37.4- 2.5) | 0.03 |
| Air_Compln | Ref | -1.7 (-22.7- 19.2) | 17.4 (-0.3- 35.0) | 26.5 (7.6- 45.3) * | 0.00 |
| Assault | Ref | -11.1 (-31.1- 9.0) | -13.3 (-33.4- 6.7) | -15.9 (-38.5- 6.6) | 0.18 |
| Burglary | Ref | -8.8 (-28.5- 10.9) | -19.0 (-40.1- 2.0) | -2.3 (-22.6- 18.0) | 0.61 |
| Crowding | Ref | -4.2 (-23.1- 14.6) | -15.5 (-34.9- 3.8) | 4.6 (-20.4- 29.5) | 0.95 |
| Delay_rent | Ref | -28.7 (-46.4- -10.9) ** | -16.3 (-34.5- 1.8) | -21.7 (-41.6- -1.8) * | 0.11 |
| F1 | Ref | -7.8 (-28.1- 12.4) | -22.2 (-43.3- -1.1) * | -17.1 (-39.7- 5.5) | 0.08 |
| F2 | Ref | -10.3 (-30.3- 9.7) | -5.4 (-26.6- 15.7) | 14.4 (-4.9- 33.7) | 0.14 |
| F3 | Ref | 0.7 (-18.3- 19.7) | 12.6 (-9.6- 34.8) | 5.0 (-17.1- 27.2) | 0.46 |
| Food_Stamps | Ref | -10.7 (-28.8- 7.4) | -29.3 (-49.6- -9.0) ** | -16.2 (-35.0- 2.6) | 0.04 |
| Housing_Violation | Ref | -18.7 (-37.4- 0.0) | -22.0 (-40.6- -3.4) * | -19.1 (-41.3- 3.0) | 0.10 |
| Larceny | Ref | -2.6 (-23.2- 18.0) | -3.4 (-26.4- 19.7) | 6.4 (-14.5- 27.3) | 0.59 |
| Murder | Ref | -16.7 (-34.7- 1.3) | -29.2 (-47.4- -11.0) ** | -22.1 (-42.4- -1.8) * | 0.02 |
| No_Doctor | Ref | -4.6 (-24.2- 15.1) | 0.8 (-20.3- 22.0) | 8.6 (-13.6- 30.9) | 0.39 |
| No_Insurance | Ref | -4.2 (-23.7- 15.2) | -15.5 (-36.4- 5.4) | -3.3 (-25.1- 18.5) | 0.55 |
| No_Med_Care | Ref | -18.1 (-38.8- 2.7) | -6.2 (-26.1- 13.7) | -11.4 (-32.8- 10.1) | 0.52 |
| Noise_Freq | Ref | -1.3 (-21.3- 18.7) | 10.8 (-11.0- 32.5) | 0.4 (-21.4- 22.2) | 0.71 |
| Noise_Neigh | Ref | -21.3 (-40.5- -2.0) * | -12.7 (-31.4- 6.0) | -23.4 (-44.3- -2.5) * | 0.08 |
| Unclean_Parks | Ref | -20.7 (-39.7- -1.8) * | -8.4 (-29.0- 12.2) | -23.2 (-41.7- -4.7) * | 0.07 |
| Poverty | Ref | -7.6 (-13.0- -2.1) ** | -10.5 (-16.5- -4.5) *** | -13.3 (-20.1- -6.5) *** | 0.00 |
| Public_Insur | Ref | -5.4 (-25.2- 14.3) | -14.4 (-34.3- 5.5) | 3.0 (-18.7- 24.7) | 1.00 |
| Robbery | Ref | -2.0 (-22.0- 18.1) | -12.5 (-32.6- 7.6) | -12.9 (-35.6- 9.8) | 0.19 |
| Safety | Ref | -3.9 (-23.6- 15.8) | -18.5 (-38.4- 1.3) | -11.6 (-33.8- 10.5) | 0.17 |
| Sidewalks | Ref | -8.7 (-28.0- 10.6) | -14.3 (-35.6- 7.0) | -9.0 (-31.5- 13.4) | 0.37 |
| Traffic_Noise | Ref | 4.7 (-15.0- 24.4) | 2.7 (-19.6- 25.0) | 18.2 (-1.8- 38.2) | 0.12 |
| Unemployment | Ref | 15.2 (-3.9- 34.4) | -4.7 (-23.5- 14.1) | -12.0 (-33.6- 9.6) | 0.11 |

**Supplemental Table S4:** Predicted change in birthweight per IQR increase in NO_2_, by stressor quartile, and linear p-value for trend across quartiles based on interaction models with the addition of mutually adjusting for significant stressors from Table 3. Models were adjusted for averaged prenatal PM_2.5_ exposure, maternal age, race/ ethnicity, maternal age, education, Medicaid status (yes/no), pre-pregnancy body mass index, receipt of prenatal care, year of conception, parity, infant sex, gestational age (in weeks), conception year, and season. Significant interactions (by p < .05) shown in **bold**.

| **Social Stressor** | **Quartile** | **Change**  **in birthweight (g)**  **per IQR NO_2_** | **p-trend** |
| --- | --- | --- | --- |
| **Abuse** | Q1 | -15.82 | 0.07 |
|  | Q2 | -12.28 |  |
|  | **Q3** | -61.5 |  |
|  | Q4 | -44.02 |  |
| Assault | Q1 | -15.42 | 0.65 |
|  | Q2 | 8.72 |  |
|  | Q3 | -14.33 |  |
|  | Q4 | -1.68 |  |
| Food_Stamps | Q1 | -16.24 | 0.37 |
|  | Q2 | -34.25 |  |
|  | Q3 | -37.32 |  |
|  | Q4 | -16.88 |  |
| **Murder** | Q1 | -15.01 | 0.01 |
|  | Q2 | -30.4 |  |
|  | Q3 | -33.76 |  |
|  | **Q4** | -71.03 |  |
| **Unclean_Parks** | Q1 | -16.85 | 0.004 |
|  | Q2 | -23.02 |  |
|  | **Q3** | -50.03 |  |
|  | **Q4** | -53.38 |  |
| **Poverty** | Q1 | -14.4 | 0.06 |
|  | Q2 | -23.54 |  |
|  | **Q3** | -43.14 |  |
|  | Q4 | -22.19 |  |
| Robbery | Q1 | -16.05 | 0.25 |
|  | Q2 | -6.97 |  |
|  | Q3 | -28.46 |  |
|  | Q4 | -27.05 |  |
| Safety | Q1 | -14.42 | 0.35 |
|  | Q2 | 13.85 |  |
|  | Q3 | -7.82 |  |

|  | Q4 | -11.46 |  |
| --- | --- | --- | --- |
| **Unemployment** | Q1 | -15.04 | 0.18 |
|  | Q2 | 8.96 |  |
|  | Q3 | -3.69 |  |
|  | **Q4** | -27.29 |  |

**Supplemental Table S5**. **Average predicted birthweights with 95% confidence intervals, by stressor quartile.** Estimates based on continuous-categorical NO_2_-social stressor interaction models across quartiles.

| Social Factor Indicator | Q1 | Q2 | Q3 | Q4 |
| --- | --- | --- | --- | --- |
| Abuse | 3404.8 (3379.9- 3429.6) | 3391.2 (3360.9- 3421.5) | 3348.3 (3319.5- 3377.0) | 3368.2 (3336.4- 3400.1) |
| Air_Compln | 3381.6 (3354.7- 3408.4) | 3364.9 (3331.6- 3398.2) | 3408.0 (3378.2- 3437.9) | 3395.0 (3366.4- 3423.6) |
| Assault | 3403.7 (3377.9- 3429.5) | 3394.1 (3362.3- 3426.0) | 3362.6 (3335.7- 3389.5) | 3354.8 (3317.2- 3392.5) |
| Burglary | 3385.2 (3356.4- 3413.9) | 3373.4 (3344.6- 3402.3) | 3381.8 (3352.8- 3410.9) | 3378.8 (3348.5- 3409.2) |
| Crowding | 3395.3 (3371.1- 3419.6) | 3363.4 (3335.2- 3391.6) | 3367.6 (3337.9- 3397.4) | 3398.1 (3357.1- 3439.0) |
| Delay_rent | 3402.7 (3378.9- 3426.5) | 3374.3 (3344.1- 3404.5) | 3375.7 (3343.9- 3407.4) | 3368.4 (3337.0- 3399.8) |
| F1 | 3401.2 (3373.4- 3429.0) | 3394.1 (3367.2- 3421.0) | 3356.9 (3327.9- 3386.0) | 3357.6 (3322.9- 3392.2) |
| F2 | 3386.8 (3360.9- 3412.7) | 3393.4 (3361.2- 3425.6) | 3365.3 (3331.7- 3398.9) | 3394.4 (3366.0- 3422.8) |
| F3 | 3386.1 (3362.1- 3410.2) | 3369.4 (3339.5- 3399.3) | 3381.3 (3346.5- 3416.0) | 3387.9 (3354.1- 3421.7) |
| Food_Stamps | 3406.6 (3382.6- 3430.6) | 3367.7 (3340.0- 3395.3) | 3361.3 (3330.9- 3391.6) | 3366.9 (3334.4- 3399.4) |
| Housing_Violation | 3403.3 (3379.1- 3427.6) | 3389.0 (3359.0- 3418.9) | 3353.3 (3323.0- 3383.6) | 3360.7 (3325.8- 3395.5) |
| Larceny | 3376.9 (3348.1- 3405.8) | 3386.9 (3358.3- 3415.4) | 3410.9 (3374.1- 3447.8) | 3378.6 (3350.7- 3406.4) |
| Murder | 3405.2 (3381.0- 3429.3) | 3376.2 (3344.4- 3408.1) | 3381.5 (3353.0- 3409.9) | 3345.4 (3312.6- 3378.2) |
| No_Doctor | 3382.6 (3357.2- 3408.0) | 3384.2 (3355.8- 3412.6) | 3377.3 (3344.8- 3409.8) | 3384.8 (3350.7- 3418.9) |
| No_Insurance | 3388.9 (3364.8- 3413.1) | 3382.3 (3351.8- 3412.8) | 3358.4 (3325.5- 3391.3) | 3387.6 (3353.8- 3421.3) |
| No_Med_Care | 3392.1 (3366.7- 3417.6) | 3379.1 (3348.7- 3409.5) | 3396.9 (3366.1- 3427.6) | 3354.3 (3321.0- 3387.6) |
| Noise_Freq | 3381.8 (3354.3- 3409.3) | 3384.1 (3354.9- 3413.4) | 3391.6 (3360.8- 3422.3) | 3376.1 (3343.1- 3409.0) |
| Noise_Neigh | 3396.3 (3371.3- 3421.2) | 3392.6 (3362.9- 3422.3) | 3370.2 (3339.8- 3400.5) | 3364.3 (3331.4- 3397.2) |
| Poverty | 3390.9 (3371.2- 3410.7) | 3368.6 (3344.9- 3392.2) | 3349.5 (3323.3- 3375.7) | 3372.1 (3344.5- 3399.7) |
| Public_Insur | 3392.1 (3368.2- 3416.0) | 3377.7 (3348.6- 3406.9) | 3371.1 (3338.5- 3403.7) | 3368.9 (3333.1- 3404.6) |
| Robbery | 3399.5 (3373.9- 3425.1) | 3388.8 (3359.7- 3417.9) | 3361.6 (3333.5- 3389.6) | 3352.9 (3315.1- 3390.7) |
| Safety | 3396.2 (3370.5- 3421.9) | 3393.1 (3364.6- 3421.7) | 3369.0 (3339.1- 3398.9) | 3347.1 (3311.2- 3382.9) |
| Sidewalks | 3391.4 (3366.9- 3416.0) | 3383.3 (3353.3- 3413.3) | 3370.7 (3338.9- 3402.4) | 3371.5 (3336.4- 3406.6) |
| Traffic_Noise | 3382.4 (3353.8- 3410.9) | 3402.9 (3375.1- 3430.7) | 3363.0 (3332.3- 3393.6) | 3392.7 (3362.5- 3422.9) |
| Unclean_Parks | 3407.4 (3383.4- 3431.3) | 3374.0 (3343.7- 3404.2) | 3352.1 (3316.0- 3388.3) | 3362.0 (3331.4- 3392.6) |
| Unemployment | 3392.6 (3366.6- 3418.5) | 3386.2 (3359.2- 3413.2) | 3387.6 (3357.9- 3417.3) | 3347.2 (3312.4- 3382.0) |

**Supplemental Figure 1.** Observed NO_2_-birthweight associations, based on GAMM interaction models with natural splines (n = 5 knots), fit separately by quartile of Stressor Factor 1 (violence and physical disorder). (Top, L to R): Q1 and Q2, (Bottom, L to R): Q3 and Q4.


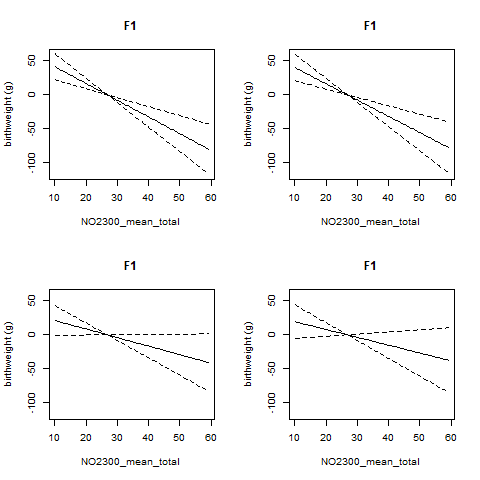


**Supplemental Figure 2.** Changes in birthweight based on GAM fits, without adjustment for clustering by UHF, across Quartiles of Factor 1 (violence and physical disorder). (Top, L to R): Q1 and Q2, (Bottom, L to R): Q3 and Q4.


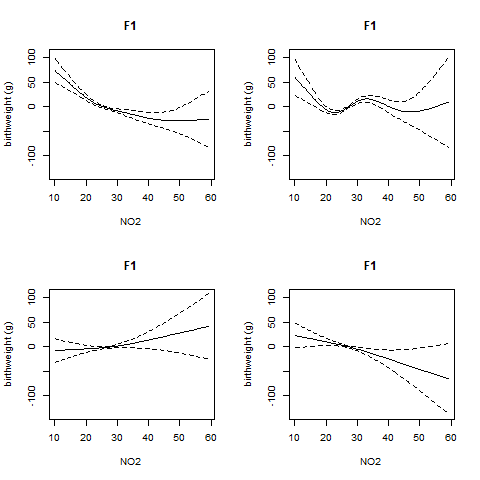

Supplement: Supplementary file 1 — Supplementary Material 1. [file 12940_2025_1189_MOESM1_ESM.docx]
